# Granulocyte Macrophage-Colony Stimulating Factor (GM-CSF) Downregulates the Expression of Protumor Factors Cyclooxygenase-2 and Inducible Nitric Oxide Synthase in a GM-CSF Receptor-Independent Manner in Cervical Cancer Cells

**DOI:** 10.1155/2015/601604

**Published:** 2015-07-15

**Authors:** Nanyan Jiang, Zhiqiang Tian, Jun Tang, Rongying Ou, Yunsheng Xu

**Affiliations:** ^1^Department of Dermatovenerology, First Affiliated Hospital of Wenzhou Medical University, Wenzhou 325000, China; ^2^Institute of Dermatovenerology, Wenzhou Medical University, Wenzhou 325000, China; ^3^Jingmen First People's Hospital, Hubei 448000, China; ^4^Institute of Immunology, PLA, Third Military Medical University, Chongqing 400038, China; ^5^Department of Dermatology, 105th Hospital of PLA, Hefei 230001, China; ^6^Department of Gynecology and Obstetrics, First Affiliated Hospital of Wenzhou Medical University, Wenzhou 325000, China

## Abstract

Enhanced expression of cyclooxygenase-2 (COX-2) and inducible nitric oxide synthase (iNOS) is associated with the pathogenic processes of various tumor types. COX-2 and iNOS expression in the immunomodulatory dendritic cells is mediated by the granulocyte macrophage-colony stimulating factor (GM-CSF), which is also expressed by cervical cancer cells; however, whether and how GM-CSF regulates COX-2 and iNOS expression in clinical cervical cancer cells remain unknown. In this study, we found that the COX-2 and iNOS expression was upregulated in the cervical cancer tissues and positively correlated with cancer metastasis and stage. About one-half of the cervical cancer tissues showed strong/moderate GM-CSF expression, while the normal cervical tissues showed >80% positive rate; no GM-CSFR protein was detectable on the cervical cancer cells. The GM-CSF expression was negatively correlated with the COX-2 and iNOS expression in the cervical cancer tissues and the functional negative regulatory effect of GM-CSF on COX-2/iNOS expression was demonstrated in various cervical cancer cell lines. Therefore, in cervical cancer cells, GM-CSF might contribute an antitumor response by inhibiting iNOS and COX-2 expression in a GM-CSFR independent manner.

## 1. Backgrounds

Cervical carcinoma is one of the most common gynecologic cancers diagnosed and represents a substantial threat to the wellbeing and survival of the world's adult female population. While human papillomavirus (HPV) infection (both chronic and persistent) of the cervical epithelium cells is recognized as the major risk factor of cervical cancer, it is not sufficient for cancer occurrence, since most women with HPV infection do not develop cervical cancer [1, 2].

The tumorigenic process of cervical cancer involves an array of immunomodulatory cytokines as well, including interleukins (IL) 6, 10, and 12 and transforming growth factor- (TGF-)*β* [3, 4], all of which are expressed by tumor cells. Cervical cancer cells have also been shown to secrete the granulocyte macrophage-colony stimulating factor (GM-CSF) [5, 6], an important hematopoietic cytokine that is known to contribute to the survival, proliferation, and differentiation of bone marrow hematopoietic cells and to function as a cofactor to influence differentiated cells of other lineages and hematopoietic multipotent precursor cells [7]. In the immune response, GM-CSF helps to regulate the response to infection and inflammatory signaling cascades by recruiting dendritic cells and triggering their maturation as well as promoting cell-mediated immunity pathways [8]. Nevertheless, the immunological effect of cervical cancer-derived GM-CSF in the cervical cancer environment is still poorly understood. In addition, the GM-CSF receptor (GM-CSFR) is expressed by many of the mature hematopoietic cell types, including neutrophils, monocytes, and eosinophils [9], as well as some nonhematopoietic cells, such as the small cell carcinoma lines and human prostate cancer cells [9, 10]. However, whether cervical cancer cells express GM-CSFR and whether the GM-CSF/GM-CSFR signaling pathway contributes to the pathogenic process of cervical cancer remain unknown.

Human carcinomas are also associated with upregulation of the inducible nitric oxide synthase (iNOS) and cyclooxygenase-2 (COX-2), which are otherwise generally not expressed in normal (noncancerous) tissues, with the exception of placenta, kidney, and brain [11, 12]. Human carcinomas exhibiting high levels of COX-2 and iNOS expression include those in stomach, liver, lung, pancreas, colon, and cervix [13–15]. These two synthases are involved in many physiological and pathological processes, and their combined expression is closely related to the tumor biological behaviors of growth, progression, metastasis, and prognosis [14]. A key function of iNOS is the enzymatic conversion of arginine to generate a locally high concentration of nitric oxide (NO) [13]; from a tumorigenic perspective, the iNOS-mediated increase in NO supports cancer development [15]. On the other hand, COX-2 acts as one of the rate-limiting enzymes in the metabolic pathway that produces eicosanoids; from a tumorigenic perspective, the PTGS2 gene encoding COX-2 acts as an early response gene inducible by carcinogens, tumor promoters, and oncogenes [2]. The demonstrated correlation of COX-2 expression with several types of tumors has suggested potential roles for this enzyme in cancer progression, specifically by inducing proliferation, enhancing mitogenesis, reducing cellular adhesion, and aiding in tumor escape from immune surveillance [16].

The above evidence indicates that cervical cancer cells express GM-CSF and synthases COX-2/iNOS. Intriguingly, it has been reported that GM-CSF can upregulate COX-2 and iNOS expression in skin dendritic cells [17]. However, whether and how GM-CSF regulates COX-2 and iNOS expression in human cervical cancer tissues remain to be clarified yet. To this end, this study was designed to determine whether GM-CSF plays a role in human cervical cancer via influence on iNOS/COX-2 and to investigate the related underlying mechanism; the results from this study were expected to not only provide insight into the pathogenic processes of cervical cancer but identify molecular factors representing potential therapeutic targets.

## 2. Materials and Methods

### 2.1. Cervical Cancer Tissues

Eighty-seven specimens of clinical stage Ib-IIb squamous cell carcinoma and 24 specimens of cervical intraepithelial neoplasia (CIN) were obtained from adult female patients (age range: 31–68 years, median: 46.51 years) who had undergone radical hysterectomy with lymphadenectomy. Sixteen normal cervical tissues were obtained from women who had undergone hysterectomy for nonneoplastic indications and had normal findings from the last cervical smear. All specimens were obtained with written informed consent from each study participant. The tissue samples were fixed in 4% buffered paraformaldehyde, embedded in paraffin, and stained with hematoxylin and eosin for histological analysis. The CIN and primary cervical cancer specimens were graded according to the WHO criteria (CIN1, CIN2, and CIN3 classifications) and the International Federation of Gynecology and Obstetrics (FIGO) criteria (Ib_1_, Ib_2_, IIa, or IIb cervical carcinoma stages). The study was approved by the Ethics Committee of Southwest Hospital for Clinical Investigation (Chongqing, China).

### 2.2. Immunohistochemistry (IHC) Assay

The paraffin-embedded tissue samples were sectioned (5 *μ*m thickness) and mounted on glass slides for IHC analysis according to the published staining methods [18]. Briefly, after blocking of nonspecific sites (5% bovine serum albumin, room temperature, 30 min), the slides were incubated (4°C, overnight) with the primary antibodies to mouse monoclonal anti-human GM-CSF (1 : 30 dilution; R&D Systems, Wiesbaden, Germany), mouse monoclonal anti-human GM-CSF receptor *α* or *β* (1 : 50; Santa Cruz Biotechnology, Dallas, TX, USA), rabbit anti-iNOS (1 : 100; Boster, Wuhan, Hubei, China), and rabbit anti-COX-2 (1 : 100; Boster), followed by incubation (4°C, overnight) with the appropriate secondary antibodies. Staining with diaminobenzidine (DAB) tetrahydrochloride was performed to localize antigens, and the sections were further counterstained with Mayer's hematoxylin. After dehydration, a protective coverslip was manually mounted.

### 2.3. Assessment of IHC Slide

A pathologist who was blinded to the clinical features of the study participants evaluated the processed samples. GM-CSF, iNOS, and COX-2 immunoreactivity was graded according to the German Immunoreactive scoring system [19], which involves rating the staining intensity in the cytoplasm on a scale from 0 to 3 (0: none, 1: weak, 2: moderate, and 3: strong). The numbers of positive and negative cells were counted (with no less than 500 cells considered as the minimum acceptable count number) and used to calculate the percentage of positive cells for scoring as follows: 0, no staining; (1), 1–10%; (2), 11–50%; (3), 51–80%; and (4), 81–100%. The final immunoreactivity score was calculated by multiplying the staining intensity score by the score for positive cells percentage, with a range from 0 to 12 whereby 0 indicated no immunoreactivity, 1–4 indicated weak immunoreactivity, 5–8 indicated moderate immunoreactivity, and 9–12 indicated strong immunoreactivity. For statistical purposes, the final immunoreactivity scores were grouped as negative/weak immunoreactivity and moderate/strong immunoreactivity [20].

### 2.4. Human Cervical Cancer Cell Lines, DNA Transfection, and siRNA Knockdown

The Hela (HPV-18-infected), SiHa, and Ca Ski (with both of the latter being HPV-16-infected) human cervical cancer cell lines were purchased from the China Center for Type Culture Collection (Wuhan, Hubei, China). All cell lines were grown in RPMI 1640 medium (HyClone, Logan, UT, USA) supplemented with l0% heat-inactivated fetal calf serum (HyClone) and 1% penicillin and streptomycin (Beyotime, Shanghai, China) at 37°C in a 5% CO_2_ atmosphere.

To establish overexpression of GM-CSF in the cervical cancer cell lines, the hGM-CSF-expressing plasmid pcDNA3.1(−)/GM-CSF (hereafter referred to as pGM-CSF) and the vector pcDNA3.1(−) were purchased from Invitrogen (San Diego, CA, USA) and lipid-mediated DNA transfection (Lipofectamine 2000; Invitrogen) was performed. To silence the expression of hGM-CSF, the cancer cell lines were seeded in 6-well tissue culture plates (10^5^ cells/mL) and transfected using the Lipofectamine 2000 Reagent to introduce 5 nM of GM-CSF siRNA (number sc39391; Santa Cruz Biotech) or scrambled control siRNA (number sc-37007, Santa Cruz Biotech). For both procedures, at 48 h postinoculation, the cells were harvested for Western blot analysis to verify the expression of hGM-CSF by using an anti-human GM-CSF antibody (R&D Systems) as the primary antibody.

### 2.5. Western Blot Assay

The levels of iNOS/COX-2 protein in the processed cancer cells were detected by Western blot as described previously [21]. Briefly, cells were washed twice with PBS and lysed in RIPA lysis buffer (Beyotime). After measurement of total protein by the bicinchoninic acid (BCA) assay, equal sample amounts were loaded for separation through a 4–12% gradient SDS-polyacrylamide gel (Invitrogen) and electrotransfer onto a polyvinylidene fluoride membrane. The membrane was then blocked with 5% skim milk powder, followed by hybridization with the respective primary and secondary antibodies. Detection of immunoreactive bands was performed with an enhanced chemiluminescence substrate (Beyotime). Quantitative analysis was carried out with a 9-image scanner densitometer (Alpha Innotech, Santa Clara, CA, USA) and normalized to the actin control. In addition, the signals were detected by use of a Luminol chemiluminescence detection kit (Santa Cruz Biotechnology) and exposure to Hyperfilm (Amersham, Piscataway, NJ, USA). Analysis of the Western blot data was repeated at least three times.

### 2.6. Statistical Analysis

All statistical analyses were performed by using the SPSS statistical software for Windows (version 17.0; SPSS Inc., Chicago, IL, USA). The chi-square test or Fisher's exact test was used for association analysis between immunostaining results and clinical characteristics. General linear regression model was used to determine possible correlations between GM-CSF negative/weak or moderate/strong expression and COX-2/iNOS protein expression in cervical cancer tissues. Unpaired Student's *t*-test was used to evaluate the differences in COX-2 and iNOS protein expression among GM-CSF transfected cells (GT), vector control (VT), and blank control (NT). A *p* value less than 0.05 was considered statistically significant.

## 3. Results

### 3.1. Expression of COX-2, iNOS, GM-CSF, and GM-CSFR in Cervical Cancer Tissue

**Figure 1:**
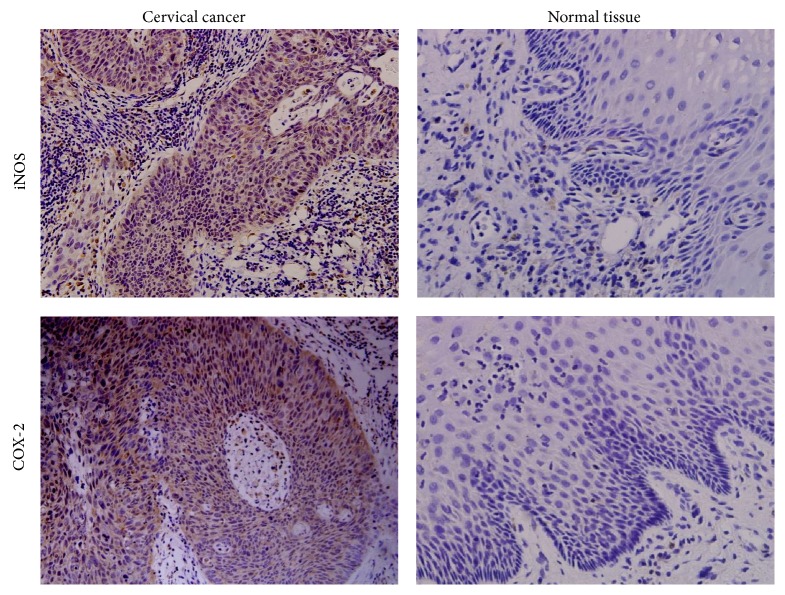
Expression of COX-2 and iNOS in cervical cancer tissues. Representative results of IHC assays showing the moderate/strong expression of COX-2 and iNOS in the cervical cancer tissues and the lack of expression in the normal cervical tissues. Brown color indicates positive staining for the indicted proteins.

As shown in Figure 1 and Table 1, the IHC assay showed positive expression of COX-2 and iNOS in 49 of 87 (56.3%) and 46 of 87 (52.9%) of the cervical cancer tissue samples, respectively. While similar positive rates for COX-2 and iNOS expression were observed in the CIN tissues, the normal cervical tissues showed no COX-2 and iNOS expression. Furthermore, the detected COX-2/iNOS expression in tumor cells was mainly localized to the cytoplasm, showing a diffuse pattern; a lower level of expression was also detected in the adjacent stromal cells of the cancer samples.

**Figure 2:**
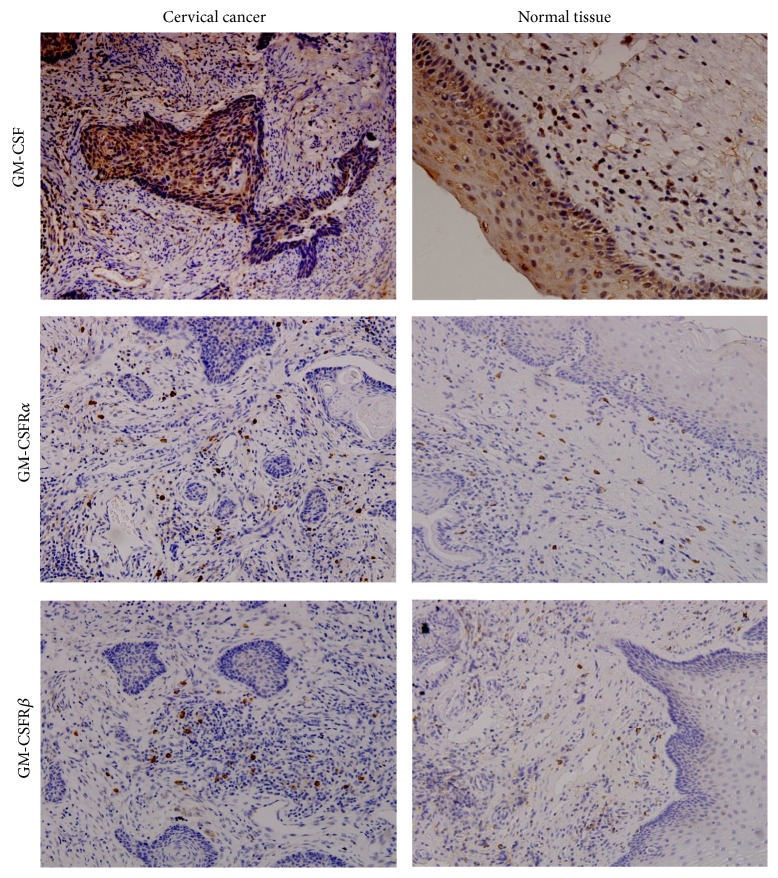
Expression of GM-CSF and GM-CSFR in cervical cancer tissues. Representative results of IHC assays showing the moderate/strong GM-CSF and GM-CSFR (*α* and *β* chains) expression observed in the cervical cancer tissues and normal cervical tissues. Brown color indicates positive staining for the indicated proteins.

Although moderate/strong expression of GM-CSF was detected in 44 out of the 87 (50.6%) cervical cancer tissues examined, this rate was markedly lower than that in the normal cervical tissues (81.3%) (Table 1 and Figure 2). In addition, expression of GM-CSFR was detected at similar levels in both cervical cancer tissues and normal cervical tissues (Figure 2); however, the GM-CSFR expression was not present in the cervical cancer nest and instead was mainly localized within the stromal cells (Figure 2).

### 3.2. Expression of COX-2/iNOS, and Not GM-CSF, Was Correlated with the Clinical Characteristics of Cervical Cancer Patients

Associations analysis showed that neither COX-2 nor iNOS expression was associated with patient age, tumor size, or HPV infection (all *p* values >0.05); however, both COX-2 and iNOS expressions were positively correlated with lymph node metastasis and FIGO stage (Table 2) (*p* < 0.05). No significant associations were found to exist between GM-CSF expression and any of the patients' clinical parameters, for either the squamous cell carcinoma or CIN patient groups (Table 2) (*p* > 0.05).

### 3.3. Expression of COX-2/iNOS Was Negatively Correlated with GM-CSF

Rank correlation analysis to assess whether the enhanced COX-2/iNOS expression in cervical cancer cells was associated with GM-CSF unexpectedly showed that the expression of GM-CSF in cancer tissues had a significantly negative correlation with COX-2 (*r* = −0.500, *p* < 0.05) and iNOS (*r* = −0.473, *p* < 0.01) (Table 3).

### 3.4. Expression of iNOS/COX-2 Was Downregulated by GM-CSF in Cervical Cancer Cells

**Figure 3:**
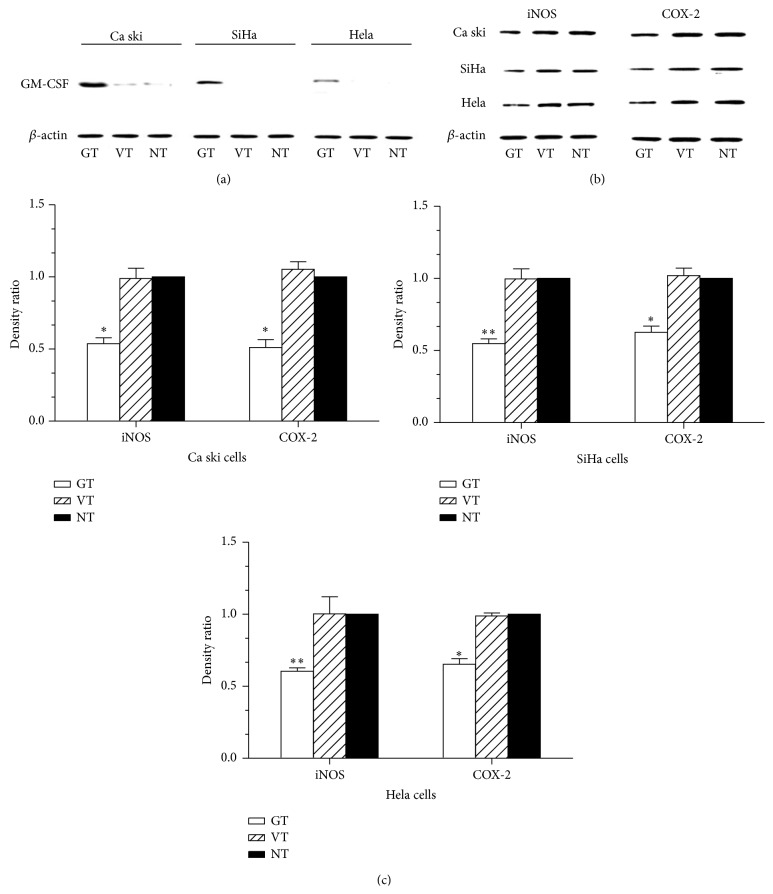
Overexpression of GM-CSF downregulated COX-2 and iNOS expression in cervical cancer cell lines. (a, b) Western blot analysis of Ca Ski, SiHa, and Hela cells untransfected or transfected with pGM-CSF or pcDNA3.1 to detect the expression of GM-CSF (a) and iNOS and COX-2 (b), respectively. (c) Unpaired Student's *t*-test was used to detect the differences of iNOS and COX-2 expression in different groups. Each experiment was repeated three times. GT: pGM-CSF transfected cells, VT: vector pcDNA3.1 transfected cells (vector control), and NT: nontreated cells (blank control). ^*∗*^ *p* < 0.05, ^*∗∗*^ *p* < 0.01 versus NT group. Error bars represent mean ± SD.

The Ca Ski cervical cancer cells were found to express a low level of endogenous GM-CSF, but the SiHa and Hela cells did not express any detectable endogenous GM-CSF (Figure 3(a)). When transfected with GM-CSF expressing plasmids, all of the cell lines showed appreciable expression of GM-CSF protein, while the cells transfected with control vectors and blank control cells did not express any detectable GM-CSF (Figure 3(a)). Notably, all of these cell lines were found to express high levels of endogenous iNOS and COX-2 protein, and this expression was found to be downregulated by about 1-fold upon pGM-CSF transfection (Figures 3(b) and 3(c)).

**Figure 4:**
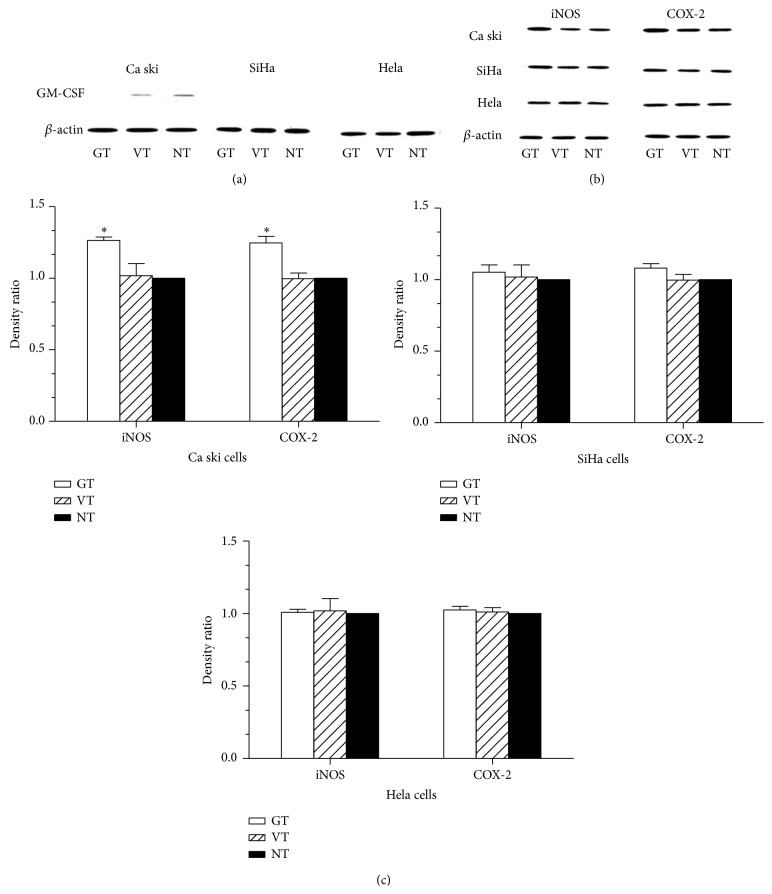
Knockdown of GM-CSF upregulates iNOS and COX-2 expression. (a, b) Western blot analysis of Ca Ski, SiHa, and Hela cells transfected with siRNA targeting the* GM-CSF* gene to detect the expression of GM-CSF (a), iNOS and COX-2 (b), respectively. (c) Unpaired Student's *t*-test was used to detect the differences of iNOS and COX-2 expression in different groups. Each experiment was repeated three times. GT: GM-CSF siRNA transfection, VT: scrambled siRNA transfection, and NT: nontransfection ^*∗*^ *p* < 0.05 versus NT group. Error bars represent mean ± SD.

Transfection of the cervical cancer cells lines with a specific siRNA sequence targeting the* GM-CSF* gene led to undetectable GM-CSF protein in the Ca Ski cells, which otherwise expressed a low level of endogenous GM-CSF protein. As expected, transfection of the vector control did not influence the GM-CSF expression, as compared with that detected in the untreated Ca Ski cells (Figure 4(a)). The SiHa and Hela cells, which did not express any detectable endogenous GM-CSF protein, showed no effect upon transfection of the siRNA sequence targeting* GM-CSF*, neither on GM-CSF expression nor iNOS and COX-2 expression (Figures 4(b) and 4(c)).

## 4. Discussion

Cervical cancer cells express synthases COX-2/iNOS and GM-CSF [5, 6, 13–15], and GM-CSF has been shown to upregulate the expression of the synthases COX-2/iNOS in skin dendritic cells [17]. However, whether and how GM-CSF regulates COX-2/iNOS expression in human cervical cancer cells remain unclear. In this study, we found that COX-2 and iNOS expression is upregulated in cervical cancer tissues, compared to normal cervical tissues, and that this upregulation is positively correlated with cancer metastasis and stage. Although about one-half of the cervical cancer tissues examined in our study showed strong/moderate GM-CSF expression, this rate was lower than that for the normal cervical tissues (which had >80% positive rate); moreover, the cervical cancer cells showed no detectable GM-CSFR expression. Our statistical analysis indicated that the GM-CSF expression detected in cervical cancer cells was negatively correlated with the COX-2 and iNOS expression in the same tissues, suggesting that the tissues with moderate/strong GM-CSF expression are prone to having less COX-2 and iNOS expression, and vice versa. The downregulating effect of GM-CSF on COX-2/iNOS expression was further confirmed in studies of three cervical cancer cell lines. Collectively, the results presented herein suggest that GM-CSF may play a protective role against cervical cancer by downregulating the protumor factors COX-2 and iNOS via a GM-CSFR-independent mechanism.

Several previous lines of evidence obtained from studies involving patients with cervical cancer have implied the clinical significance and biologic roles of both iNOS and COX-2 [18, 22–25]. A study of cervical carcinoma patients who underwent radiotherapy treatment showed that overexpression of iNOS and COX-2 was associated with greater risks of metastasis and death [14]. Another study using the Hela cell line showed associations of iNOS and COX-2 with lymph node metastasis and angiogenesis and demonstrated that the coordinated activities of these two synthases contribute to the development and occurrence of cervical cancer through their activation of the NF-*κ*B pathway [20]. Similarly, a study by Bandyopadhyay et al. [26] showed a positive association between COX-2 expression and lymph node metastasis in cervical carcinoma. These reports collectively support the proposed protumor role of COX-2 and iNOS in cervical cancer, which agrees with our observations of these two proteins being significantly enhanced in clinical samples of cervical cancer tissues and with the presumed promotion of lymph node metastasis and higher stage (more advanced) cervical cancer.

The relationship between GM-CSF and iNOS and COX-2 expression in immune cells is well documented. For instance, incubating peripheral blood mononuclear cells from a healthy donor with GM-CSF alone or in combination with the cytokines VEGF, TNF-*α*, IL-6, or IL-1*β* results in upregulation of iNOS [27]. In addition, the expression of COX-2 in activated (lipopolysaccharide- (LPS-) stimulated) monocytes may be regulated by the activities of GM-CSF-stimulated STAT5 acting in cooperation with other transcription factors such as AP-1, IL-6, and NF-*κ*B [28], while in skin dendritic cells, GM-CSF induces iNOS expression by activating the NF-*κ*B pathway [17]. However, the findings from our current study showed that GM-CSF expression is negatively correlated with the COX-2 and iNOS expression in cervical cancer tissues. Furthermore, when we established ectopic overexpression of GM-CSF in the cervical cancer cell lines Hela, SiHa, and Ca Ski, we found a marked downregulation of the expression of COX-2 and iNOS, with knockdown of GM-CSF expression leading to enhanced expression of both factors. These findings suggest that GM-CSF might exert different effects on different cell types. Specifically, the GM-CSF protein may promote the proinflammatory response in immune cells, such as APCs [29], to clear a pathogenic infection and may be useful to enhance a vaccine's efficiency by upregulating COX-2 and iNOS expression. As observed in this study, GM-CSF might suppress the expression of both protumor factors COX-2 and iNOS in tumor cells, thereby exerting an antitumor effect; therefore, the decreased GM-CSF expression in clinical cervical cancer tissues might be responsible for the upregulated expression of both protumor factors. Although the exact mechanisms underlying these potentially beneficial effects remain to be fully elucidated, the suppressive effects of GM-CSF on COX-2 and iNOS expression in cancer cells appear to occur via a GM-CSFR-independent pathway, as suggested by the lack of GM-CSFR expression in cervical cancer cells.

The occurrence of cervical carcinoma is closely related with HPV infection, and HPV may play an important role in development of the disease. It has been reported that HPV18 infection is associated with poor prognosis for patient survival and accelerated tumor growth [14]. Specific HPV proteins, such as E2, E6, and E7, have been reported as associated with certain cytokine dysfunctions, and in HPV-infected cervical epithelial cells, the HPV E2 protein has been shown to induce IL-10 activity and to cause immune suppression and persistence of the infection [30]. The downstream effects of HPV infection suggest that the presence and activity of this viral pathogen may correlate with many inflammation-related molecules, such as GM-CSF, iNOS, and COX-2. Indeed, a study has shown that iNOS expression is significantly higher in keratinocytes transduced with E6 and E7 from HPV16 than in untransduced keratinocytes [31], implying that HPV might affect expression of iNOS. However, that* in vitro* study may not fully reflect the* in vivo* effects of HPV infection on the expression of the related factors, which may explain why we did not observe any correlation between HPV infection and the expression of GM-CSF, iNOS, or COX-2 in our clinical cervical cancer tissue samples. Moreover, our finding of no HPV correlation is supported by observations from Sarian et al. [16], who demonstrated that the HPV detection rates do not differ significantly across COX-2 protein expression strata, ranging from negative to strong expression.

## 5. Conclusions

This study shows that tumor-derived GM-CSF might elicit an antitumor response in cervical cancer by inhibiting the iNOS and COX-2 expression in cervical cancer cells in a GM-CSFR-independent manner. These results suggest that GM-CSF might be a helpful adjuvant that will aid in the design of a novel cervical cancer vaccine. Further studies are necessary to explore the detailed mechanism by which GM-CSF regulates iNOS and COX-2 expression in cervical cancer cells to support the potential applications of GM-CSF in clinical cervical cancer patients.

## Figures and Tables

**Table 1:** Expression of GM-CSF, COX-2, and iNOS proteins in normal cervical, CIN, and cervical cancer tissues.

Tissue	iNOS			COX-2			GM-CSF			Total samples
	Strong/ moderate	Weak/ negative	Positive rate, %	Strong/ moderate	Weak/ negative	Positive rate, %	Strong/ moderate	Weak/ negative	Positive rate, %	
Normal cervical	0	16	0.0	0	16	0.0	13	3	81.3	16
CIN	14	10	58.3	13	11	54.2	18	6	75.0	24
Cervical cancer	46	41	52.9	49	38	56.3	44	43	50.6^∆^	87

^∆^ *p* < 0.005, cervical cancer versus normal cervical tissues. The *p* values were calculated with chi-square or Fisher's exact tests.

**Table 2:** Patient characteristics.

Characteristic	iNOS			COX-2			GM-CSF		
	Moderate/ strong (*n* = 46)	Negative/ weak (*n* = 41)	*p*	Moderate/ strong (*n* = 49)	Negative/ weak (*n* = 38)	*p*	Moderate/ strong (*n* = 44)	Negative/ weak (*n* = 43)	*p*
Age, years	46.26 ± 6.58	47.85 ± 7.66	0.304	47.18 ± 7.11	46.79 ± 7.21	0.800	47.93 ± 7.50	46.02 ± 6.62	0.213
Tumors size, cm	3.03 ± 1.21	2.86 ± 1.17	0.803	3.18 ± 1.16	2.80 ± 1.10	0.583	2.94 ± 1.01	3.02 ± 1.11	0.892
FIGO stage									
Ib1	9	17	0.027	10	16	0.019	12	14	0.983
Ib2	8	13		9	12		10	11	
IIa	15	9		16	8		12	12	
IIb	14	2		14	2		10	6	
HPV infection									
HPV(+)	34	31	0.856	38	30	0.876	29	26	0.843
HPV(−)	12	10		11	8		15	17	
LN metastasis									
Yes	32	20	0.048	33	17	0.034	26	17	0.068
No	14	21		16	21		18	26	

Data are presented as mean ± SD. The *p* values were calculated with chi-square or Fisher's exact tests.

**Table 3:** Correlation between the expression of GM-CSF and COX-2 or iNOS in cervical cancer tissues.

Index	Expression	COX-2^*∗*^		iNOS^*∗∗*^	
		Moderate/strong	Negative/weak	Moderate/strong	Negative/weak
GM-CSF	Moderate/strong	14	30	13	31
	Negative/weak	35	8	33	10

A generalized linear model was used to determine possible correlations between GM-CSF expression and COX-2/iNOS protein expression. ^*∗*^ *r* = −0.50, *p* < 0.05; ^*∗∗*^ *r* = −0.47, *p* < 0.01.
